# Characteristics of a broad lytic spectrum endolysin from phage BtCS33 of *Bacillus thuringiensis*

**DOI:** 10.1186/1471-2180-12-297

**Published:** 2012-12-19

**Authors:** Yihui Yuan, Qin Peng, Meiying Gao

**Affiliations:** 1 Key Laboratory of Agricultural and Environmental Microbiology, Wuhan Institute of Virology, Chinese Academy of Sciences, Wuhan, 430071, P.R. China

**Keywords:** Bacillus thuringinesis, Bacteriophage, Endolysin, N-acetylmuramoyl-L-alanine amidase

## Abstract

**Background:**

Endolysins produced by bacteriophages lyse bacteria, and are thus considered a novel type of antimicrobial agent. Several endolysins from *Bacillus* phages or prophages have previously been characterized and used to target *Bacillus* strains that cause disease in animals and humans. *B. thuringiensis* phage BtCS33 is a *Siphoviridae* family phage and its genome has been sequenced and analyzed. In the BtCS33 genome, *orf18* was found to encode an endolysin protein (PlyBt33).

**Results:**

Bioinformatic analyses showed that endolysin PlyBt33 was composed of two functional domains, the N-terminal catalytic domain and the C-terminal cell wall binding domain. In this study, the entire endolysin PlyBt33, and both the N- and C-termini,were expressed in *Escherichia coli* and then purified. The lytic activities of PlyBt33 and its N-terminus were tested on bacteria. Both regions exhibited lytic activity, although PlyBt33 showed a higher lytic activity than the N-terminus. PlyBt33 exhibited activity against all *Bacillus* strains tested from five different species, but was not active against Gram-negative bacteria. Optimal conditions for PlyBt33 reactivity were pH 9.0 and 50°C. PlyBt33 showed high thermostability, with 40% of initial activity remaining following 1 h of treatment at 60°C. The C-terminus of PlyBt33 bound to *B. thuringiensis* strain HD-73 and *Bacillus subtilis* strain 168. This cell wall binding domain might be novel, as its amino acid sequence showed little similarity to previously reported endolysins.

**Conclusions:**

PlyBt33 showed potential as a novel antimicrobial agent at a relatively high temperature and had a broad lytic spectrum within the *Bacillus* genus. The C-terminus of PlyBt33 might be a novel kind of cell wall binding domain.

## Background

Endolysins are enzymes produced by bacteriophages (phages) at the end of their life cycles to lyse the cell walls of host cells and release mature progeny phage particles [1,2]. Most endolysins require a second phage protein, holin, to create pores in the cytomembrane and enable them to pass through to reach their substrate, a cell wall peptidoglycan [3,4]. Because of their potential as novel antibacterial agents, the characteristics of several endolysins have previously been studied [5-10].

Endolysins of phages isolated from Gram-positive bacteria typically contain two functional domains, the N-terminal catalytic domain and the C-terminal cell wall binding domain [1]. The catalytic domain belongs to one of the four families of peptidoglycan hydrolases, which are classified according to catalytic site-specificity: *N-*acetylglucosaminidases, *N-*acetylmuramidases (lysozymes), *N-*acetylmuramoyl-L-alanine amidases, and endopeptidases [1,11]. By contrast, the cell wall binding domain is divergent and can distinguish discrete cell wall epitopes. Usually, one cell wall binding domain determines the endolysin strain specificity [11,12]; however, there are sometimes more than one [7,13,14] or even no cell wall binding domains [15,16]. The endolysin C-terminus nevertheless sometimes appears to be essential for catalytic activity, as several reports showed that the enzymatic activity is abolished after removal of the C-terminus [17,18].

*Bacillus thuringiensis* belongs to the *Bacillus cereus* group, which includes two very closely related species: *B. cereus* and *Bacillus anthracis*[19]*. B. thuringiensis* is an insect pathogen that forms an insecticidal crystal protein during sporulation [20]. *B. anthracis* is the anthrax pathogen, while *B. cereus* is a food contaminant [19]. Because of the multidrug resistance of *B. anthracis*[21,22], several of its phage or prophage endolysins have been expressed, purified, and characterized. There have also been some attempts to use these endolysins to cure the disease caused by *B. anthracis*[8,9,11,17,18,23]. Practical applications of endolysins were enabled by studies on functional domain composition, optimal reaction conditions, and species- or strain-specificity. For example, combining the catalytic domain of one endolysin with the cell wall binding domain of another changed the specificity or activity [24].

Until now, only two bacterial cell wall hydrolases from *B. thuringiensis* phage GIL01 have been reported [25], and little is known about their functional domain composition. The lytic activity of one of these hydrolases was limited to *B. thuringiensis israelensis*, while the other exhibited a broader cleavage spectrum in lysing two other Gram-positive species, *B. subtilis* and *Micrococcus lysodeikticus*.

Phage BtCS33 is a *Siphoviridae* family member that was isolated from *B. thuringiensis kurstaki* strain CS-33 [26]. The BtCS33 genome has been sequenced and a potential endolysin gene, *orf*18, was identified using bioinformatics. The gene product was named PlyBt33. In this study, we analyzed the functional domain composition of PlyBt33 using bioinformatics, and then demonstrated its biological activity after separately expressing the catalytic and cell wall binding domains in *Escherichia coli*. PlyBt33 showed a broad lytic spectrum against the tested *Bacillus* strains. Additionally, its cell wall binding domain exhibited low amino acid sequence similarity to previously reported domains.

## Results

### Identification and domain composition of endolysin from phage BtCS33

**Figure 1:**
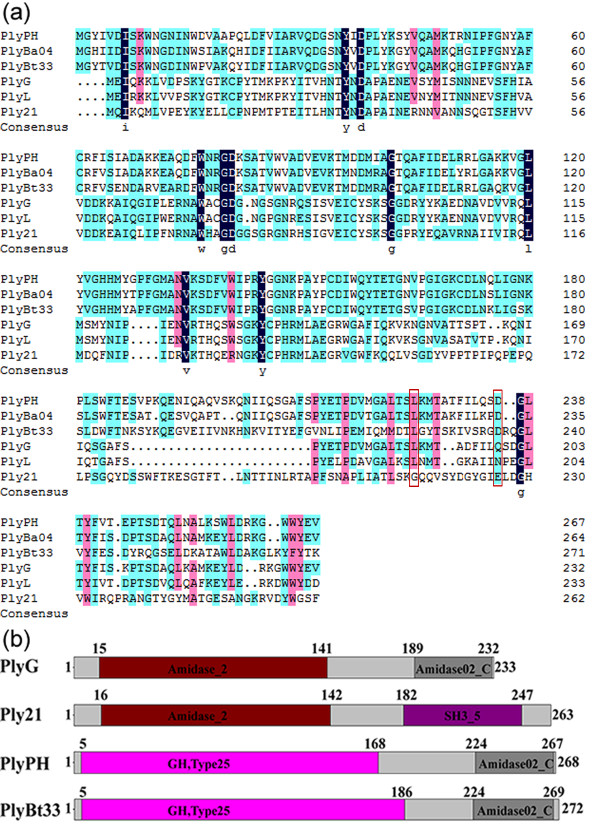
**Amino acid sequence alignment and structural composition of the studied *Bacillus *endolysins.(a)** Alignment of the amino acid sequences of PlyBt33 with other bacteriophage endolysins. PlyPH, PlyBa04, and PlyL were the putative *B. anthracis* prophage endolysins [9,16,22]; PlyG was the endolysin from *B. anthracis* phage Gamma [17,28]; Ply21 was the endolysin from *B. cereus* phage TP21[9,29]. Residues critical for the cell wall binding activity of PlyG to *B. anthracis*[30] and the corresponding residues in the other endolysins were boxed in red. **(b)** Schematic representation of PlyBt33 and other *Bacillus. sp.* endolysins. Amidase_2 and GH-25 represented the catalytic region of each endolysin; Amidase02_C and SH3_5 represented the cell wall binding region of each endolysin. The numbers above the rectangles corresponded to amino acid residue positions.

Position-specific iterated BLAST (PSI-BLAST) analysis of the phage BtCS33 genome identified *orf*18 as the gene encoding the endolysin PlyBt33. Amino acid sequence alignment of PlyBt33 with several endolysins from *Bacillus* phages or prophages (Figure 1a) revealed high similarity to PlyPH [9] and PlyBa04 [23] (about 67% and 71%, respectively), but low similarity to PlyG [18], PlyL [17], and Ply21 [27] (less than 15%).

Pfam and CDD analysis showed that PlyBt33 was composed of two functional domains (Figure 1b), the N-terminal catalytic domain (amino acid residues 5–186) and the C-terminal cell wall binding domain (amino acid residues 224–269). Figure 1b showed the Pfam analysis of four endolysins from *Bacillus* phages, and indicated that the N-terminus of PlyBt33 was a GH25 family hydrolase domain, while the C-terminus was an amidase02_C domain. PlyBt33 exhibited the same domain composition as PlyPH, but differed from PlyG and Ply21. According to homology-based endolysin classification [1], PlyBt33 is a putative member of the *N-*acetylmuramoyl-L-alanine amidases.

### Expression and purification of endolysin

**Figure 2:**
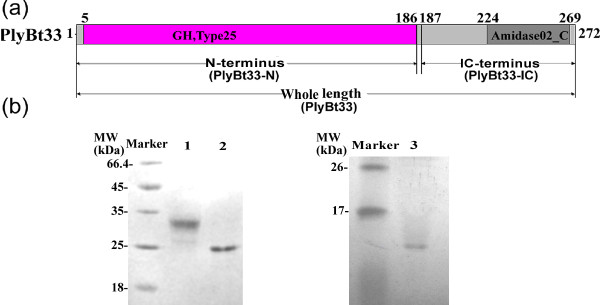
**Schematic diagram and SDS-PAGE analysis of expressed PlyBt33 and its functional domains.(a)** Schematic diagram of expressed PlyBt33 (full length), PlyBt33-N (N-terminal), and PlyBt33-IC (IC-terminal) proteins. The numbers above the rectangle correspond to amino acid residues. **(b)** SDS-PAGE analysis of expressed and purified PlyBt33, PlyBt33-N, and PlyBt33-IC proteins. Marker, molecular mass marker; lane 1, Ni-NTA column-purified PlyBt33 from *E. coli* supernatant following ultrasonication; lane 2, Ni-NTA column-purified PlyBt33-N from *E. coli* supernatant following ultrasonication; lane 3, Ni-NTA column-purified PlyBt33-IC from *E. coli* supernatant following ultrasonication. PlyBt33, PlyBt33-N, and PlyBt33-IC bands appeared at 33 kDa, 24 kDa, and 11 kDa, respectively.

To determine the function of the entire PlyBt33 protein, the N-terminal region (PlyBt33-N, amino acids 1–186), and the C-terminus combined with the internal region (PlyBt33-IC, amino acids 187–272) (Figure 2a), we constructed three recombinant strains and induced protein expression with isopropyl-β-D-thio-galactoside (IPTG). Sodium dodecyl sulfate polyacrylamide gel electrophoresis (SDS-PAGE) analysis showed that the three observed molecular weights coincided with their theoretical weights of 31 kDa, 24 kDa, and 11 kDa, respectively. SDS-PAGE analysis also showed that the purity of each protein following Ni-NTA purification exceeded 90% (Figure 2b).

### Lytic activity of PlyBt33

**Figure 3:**
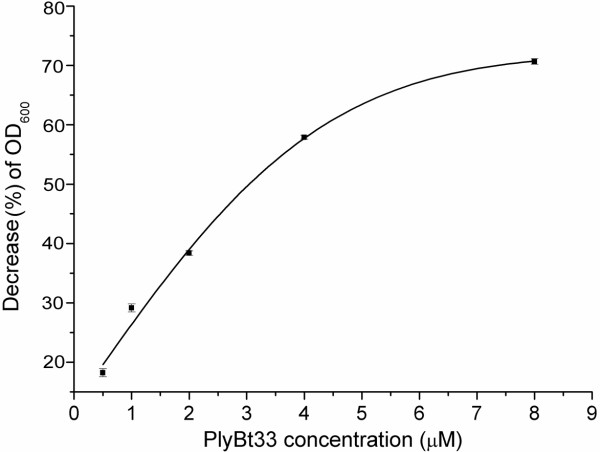
**Relationship between PlyBt33 concentration and lytic activity.** Lytic activities of PlyBt33 on viable cells of *B. thuringiensis* strain HD-73 with different PlyBt33 concentrations were tested. The initial OD_600_ of the strain suspension was 0.8 and the test was carried out at 37°C in 20 mM Tris-HCl (pH 8.0). The decrease of OD_600_ (%) = (1− the absorbance of the bacterial suspension at the end of each treatment / the absorbance at the beginning of each treatment) × 100%. The assay was carried out in triplicate and the mean values were used.

**Figure 4:**
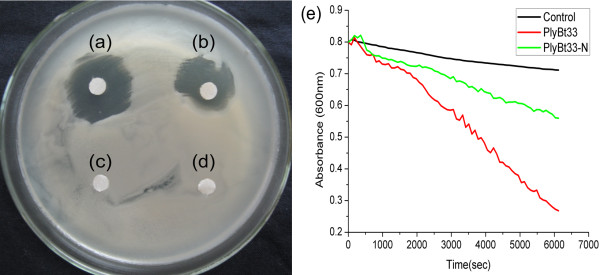
**Lytic activity assay of PlyBt33, PlyBt33-N, and PlyBt33-IC.(a)**, **(b)**, **(c)**, and **(d)**. Filter papers were soaked in the crude extract suspended in 20 mM Tris-HCl (pH8.0) of PlyBt33 **(a)**, PlyBt33-N **(b)**, and PlyBt33-IC **(c)** from *E. coli* M15, and *E. coli* M15 containing pQE-30 **(d)**, and placed onto the bacterial lawn of *B. thuringiensis* HD-73. **(e)** Lysis of viable cells using purified PlyBt33 and PlyBt33-N. Tests were performed in 20 mM Tris-HCl with a final protein concentration of 2 μM at 37°C. Crude extract of *E. coli* M15 containing pQE-30 was used as a control to treat *B. thuringiensis* strain HD-73.

**Figure 5:**
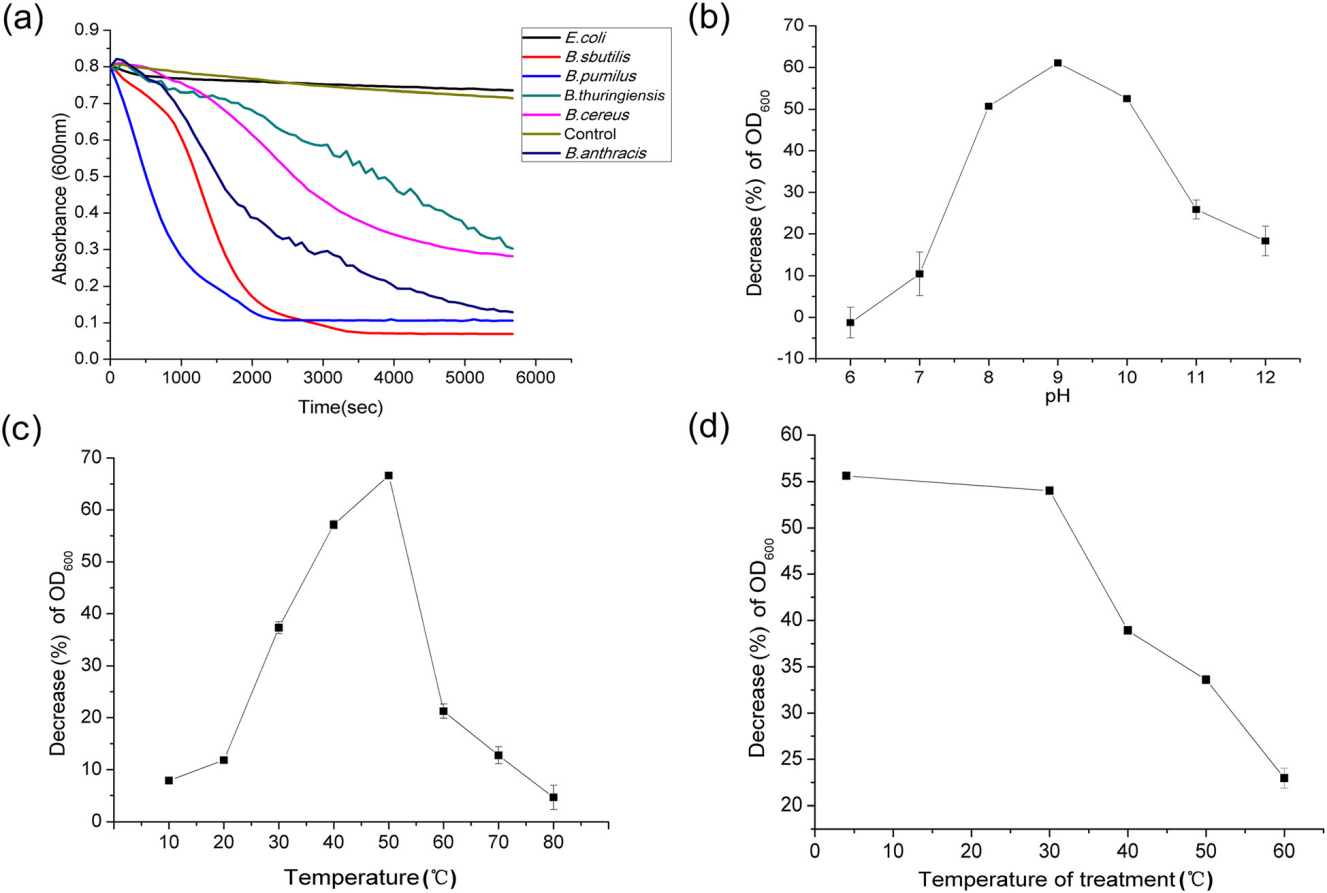
**Characterization of the endolysin PlyBt33.(a)** Lysis of viable cells from five different *Bacillus* species and one *E. coli* strain by PlyBt33. Tests were carried out with a final protein concentration of 2 μM at 37°C in 20 mM Tris-HCl (pH 8.0). The initial OD_600_ of each strain suspension was 0.8. Crude extract of *E. coli* M15 containing pQE-30 was used as a control to treat *B. thuringiensis* strain HD-73. **(b)** pH-dependent activity of PlyBt33. Tests were carried out with a final protein concentration of 2 μM at 37°C in 20 mM Tris at varying pH levels. **(c)** Temperature-dependent activity of PlyBt33. Tests were carried out with a final protein concentration of 2 μM in 20 mM Tris-HCl (pH 8.0) at varying temperatures. **(d)** Temperature stability of PlyBt33. Proteins were first treated at different temperatures for 1 h and then the tests were carried out with a final protein concentration of 2 μM at 37°C in 20 mM Tris-HCl (pH 8.0). In **(b)**, **(c)**, and **(d)**, decrease of OD_600_ (%) = (1− the absorbance of the bacterial suspension at the end of each treatment / the absorbance at the beginning of each treatment) × 100%.

The relationship between different concentrations of PlyBt33 and their corresponding lytic activities was tested. Figure 3 showed a linear relationship from 0.5 μM to 4 μM. For further assays, we used a final concentration of 2 μM as this concentration lies within the linear activity range of PlyBt33. The lytic activities of PlyBt33-N and PlyBt33-IC were investigated to determine the active region of PlyBt33. The results revealed that PlyBt33-N but not PlyBt33-IC lysed *B. thuringiensis* strain HD-73 (Figure 4a-d). This suggested that the active region of PlyBt33 was the N-terminus, although the lytic activity of PlyBt33-N was relatively low when compared with PlyBt33 (Figure 4e). To detect the lytic spectrum of PlyBt33, the lytic activity of purified PlyBt33 was tested against *B. thuringiensis* strains HD-73, HD-1, four *B. thuringiensis* isolates, *B. subtilis*, *B. pumilus*, *B cereus*, *B. anthracis,* and the Gram-negative strains *P. aeruginosa*, *Y. pseudotuberculosis*, and *E. coli.* PlyBt33 lysed all *Bacillus* strains tested, but not the Gram-negative strains. The lytic activity against *B. thuringiensis* was low, but was much higher against *B. subtilis* and *B. pumilus* (Figure 5a), which corresponded with previous reports [17,31]. Furthermore, PlyBt33 lysed *B. cereus* and *B. anthracis* with higher lytic activity.

The effects of pH and temperature on PlyBt33 lytic activity were investigated. Lytic activity against the tested strains was observed in the pH range of 7.0–12.0, with an optimal pH of 9.0 (Figure 5b). The optimum reaction temperature was 50°C (Figure 5c), and lytic activity gradually decreased as temperature increased from 30–60°C (Figure 5d). Following treatments at 40°C and 60°C for 1 h, lytic activity was reduced by 40% and 60%, respectively.

### Cell wall binding activity of PlyBt33-IC

According to previous reports, the C-termini of several characterized Gram-positive endolysins comprised one or several SH3 family cell wall binding domains [11,14,30]. Pfam analysis of PlyBt33 showed that the PlyBt33 C-terminus consisted of an Amidase02_C domain, which was present in several endolysins [9,18]. We aligned the PlyBt33 C-terminus with other characterized cell wall binding domains from *Bacillus* phage or prophage endolysins, and observed limited similarity. However, the highest similarity was found with the C-termini of PlyG, PlyL, PlyBa04, and PlyPH (Figure 1). Kikkawa *et al*. previously reported that amino acid residues L190 and Q199 of endolysin PlyG were critical for the cell wall binding activity of PlyG to *B. anthracis*[32]. Figure 1 showed that PlyBt33 and PlyG shared identical L190 residues but differred at amino acid 199.

**Figure 6:**
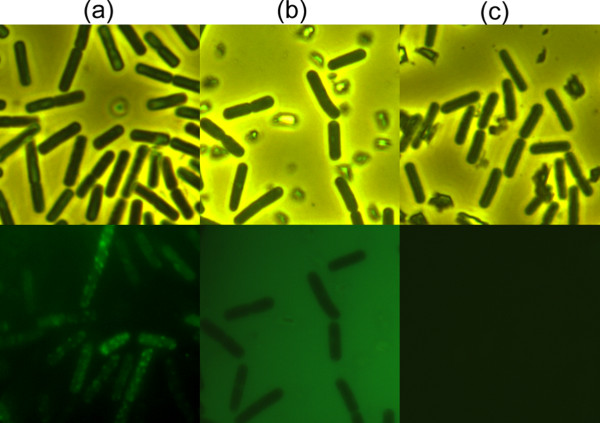
**Binding ability of FITC-PlyBt33-IC to viable cells of *B. thuringiensis *HD-73, as observed by phase contrast (upper panels) and fluorescence (lower panels) microscopy.(a)** Binding of FITC-PlyBt33-IC to the entire surface of HD-73; **(b)** No binding of FITC-BSA to HD-73 was observed; **(c)** HD-73 cell suspension with no protein was used as a control.

The PlyBt33 C-terminus was expressed, purified, and labeled with fluorescein isothiocyanate (FITC). After mixing FITC-PlyBt33-IC with the bacterial suspension for 5 min, the cells were visualized under a fluorescence microscope, and binding between FITC-PlyBt33-IC and the surface of *B. thuringiensis* HD-73 was apparent (Figure 6a). The binding ability assay was also repeated with a higher FITC-PlyBt33-IC concentration (0.05 mg/ml). At this concentration, homogenous binding of FITC-PlyBt33-IC to the cell surface was observed (data not shown), in contrast to the random binding pattern seen at the lower concentration. FITC-labeled bovine serum albumin (BSA) showed no binding to HD-73 (Figure 6b), and the HD-73 cell suspensions used as a control showed no fluorescence (Figure 6c). FITC-PlyBt33-IC also bound to *B. subtilis* 168, while no binding was detected in *E. coli* (data not shown). The binding activity of PlyBt33-IC was consistent with its lytic specificity.

## Discussion

In the present work, we expressed and determined the activity of endolysin PlyBt33 from *B. thuringiensis* phage BtCS33. The endolysin was found to be a putative *N*-acetylmuramoyl-L-alanine amidase, and was composed of an N-terminal catalytic domain and a C-terminal cell wall binding domain. PlyBt33 maintained 40% of its lytic activity against bacterial cells following treatment at 60°C for 1 h.

Though PlyBt33 exhibited a high sequence similarity (67%) to endolysin PlyPH, their characteristics were quite different. PlyPH was a *B. anthracis* putative prophage origin endolysin that could lyse *B. anthracis* and *B. cereus*, and had a broad optimal pH range (pH 4.0–10.5) [9]. By contrast, PlyBt33 exhibited lytic activity between pH 7.0–12.0, with an optimal pH of 9.0. The differences between the amino acid sequences of these two endolysins may cause differences in pI (putative pI 8.51 for PlyBt33 and 6.15 for PlyPH) and different surface net charges. Low *et al.*[23] reported that the net charge of endolysin PlyBa04 influenced its lytic activity and specificity, which might explain the different pH ranges of these two endolysins. Moreover, the lytic spectrums of PlyBt33 and PlyPH were also different. PlyBt33 could hydrolyze all tested *Bacillus* strains from five different species, while PlyPH could only lyse *B. anthracis* and *B. cereus*. Alignments of the putative cell wall binding domains of PlyBt33 and PlyPH revealed a low similarity (about 20%). According to previous reports, cell wall binding was essential in the host range determination of the endolysin [12,32,33], and this might be the cause of the different target-specificities of PlyBt33 and PlyPH. The same phenomenon was observed in endolysin PlyG (lytic specificity for *B. anthracis* and *B. cereus*) [18], which showed high similarity to PlyPH and low similarity to PlyBt33 at the putative cell wall binding region. Contrarily, *B. anthracis* endolysin PlyL showed low similarity to PlyBt33 at the putative cell wall binding region, but exhibited a relatively broad lytic spectrum. Both endolysins could lyse strains of *B. anthracis, B. cereus*, and *B. subtilis*[17]. We speculated that this was either because the different cell wall binding domains recognized the same cell wall epitope, or that there were various cell wall epitopes available for binding. Because of the low similarity of the PlyBt33 cell wall binding domain with others, we inferred that it might be a novel type of cell wall binding domain.

We observed random binding of the FITC labeled cell wall binding proteins with ligands on the cell surface (Figure 6a). The concentration used of the FITC labeled cell wall binding proteins (0.0125 mg/ml) was low, and as such only parts of the ligands were bound by the FITC labeled cell wall binding proteins. When a higher concentration (0.05mg/ml) was used, the FITC labeled cell wall binding proteins bound uniformly to the cell surface (data not shown). These results suggested a homogenous distribution of ligands on the cell surface, which agrees with the findings of previous reports [12].

In previous reports, the lytic activity of PlyL increased after removing the C-terminal region [17], while the lytic activity of PlyG was reduced [18]. Though the similarity between the N-terminal regions of PlyG and PlyL was high, they each exhibited distinct features. The similarity of PlyBt33 to PlyG and PlyL was low; therefore we decided to investigate the influence of the C-terminus on the lytic activity of PlyBt33. In this study, when the C-terminus of PlyBt33 was removed, the lytic activity was reduced. We speculated that this was due to the C-terminus assisting in the binding of PlyBt33 to the catalytic epitope on the cell wall of target bacteria, which benefits the catalysis of PlyBt33.

PlyBt33 had a relatively high thermostability, which, combined with its high lytic activity against *B. cereus* (a source of toxins in the food industry) [34,35], suggested that it had the potential to be an extremely useful antimicrobial agent in food production processes involving heat treatment [36]. PlyBt33 also exhibited a high lytic activity against *B. anthracis*, which indicated that it could be used in the treatment of anthrax [19].

## Conclusions

The endolysin PlyBt33 was composed of two functional domains, the N-terminal catalytic domain and the C-terminal cell wall binding domain. The C-terminus of PlyBt33 might be a novel kind of cell wall binding domain. PlyBt33 lysed all tested *Bacillus* strains from five different species. Optimal conditions for PlyBt33 were pH 9.0 and 50°C, and PlyBt33 was also found to be relatively thermostable. These characteristics indicated that PlyBt33 might be an extremely useful antimicrobial agent in food production processes that involve heat treatment, and in the treatment of anthrax.

## Methods

### Bacterial strains and cultures

*E. coli* expression of the endolysin gene, respectively. *B. thuringiensis* strain HD-73 is the standard strain of *B. thuringiensis subsp. kurstaki*[37], while *B. subtilis* strain 168, obtained from Dr. Yuan Zhiming (Wuhan Institute of Virology, Chinese Academy of Sciences, Wuhan, China), is the most widely used model strain of *B. subtilis*[38]. *B. anthracis* CMCC63605 with the pXO1 plasmid eliminated was provided by Dr. Yuan Zhiming (Wuhan Institute of Virology, Chinese Academy of Sciences, Wuhan, China). *B. thuringiensis* strain CS-33 (CCTCC No. M202025) and phage BtCS33 (CGMCC7.61) were isolated by our laboratory. Other *B. thuringiensis*, *B. cereus*, and *B. pumilus* strains used in this study were collected and identified by our laboratory. *Pseudomonas aeruginosa* PAO1 (ATCC47085) and *Yersinia pseudotuberculosis* NaI (provided by Dr. Wang Yao, Wuhan Institute of Virology, Chinese Academy of Sciences, Wuhan, China) were used to test the lytic spectrum of the endolysin. All strains were grown in LB medium.

### Bioinformatic analysis of the putative endolysin gene of phage BtCS33

Open reading frames (ORFs) of the phage BtCS33 genome (GenBank: JN191664) were predicted using FGENE SV software (http://linux1.softberry.com/berry.phtml?topic=virus&group=programs&subgroup=gfindv) and by visual inspection. The non-redundant protein database was searched using BLASTP [39] with the amino acid sequences of endolysins from BtCS33 and PlyBt33 as the query. ORF18 was predicted to encode the endolysin from BtCS33. Amino acid sequences of PlyBt33 and several known endolysins were aligned using ClustalW2 [40] and manually adjusted. Functional domains were searched against the Pfam database (http://pfam.sanger.ac.uk/search) [41] and the CDD database (http://www.ncbi.nlm.nih.gov/cdd) [42].

### Plasmid construction and transformation

DNA manipulations were performed according to standard protocols [43]. Phage BtCS33 genomic DNA was extracted as previously described [44] and used as a template to amplify the entire endolysin gene (ORF18, also known as *plyBt33* and expressed as protein PlyBt33), the N-terminal region gene (*plyBt33-N*, expressed as PlyBt33-N), and the internal and C-terminal region gene (*plyBt33-IC*, expressed as PlyBt33-IC). Primers and corresponding PCR products are listed in Table 1. Amplifications were performed in a Veriti 96-Well Thermal Cycler (Applied Biosystems, Foster City, CA) with an annealing temperature of 55°C. PCR products were purified using a DNA extraction kit (Omega Bio-Tek, Norcross, GA) and inserted into the *Bam*HI/*Sal*I site of pQE-30 (Qiagen, Germany), which contains a His-tag for protein purification. Three recombinant plasmids were transformed into *E. coli* TG1, and three into *E. coli* M15. Positive transformants were selected and verified by DNA sequencing using a PRISM 3730 DNA analyzer (Applied Biosystems).

**Table 1:** Oligonucleotide primers pairs used in this study

**Primer pairs**	**Sequence (5'-3')**	**PCR products (Size)**	**Predicted products/Size (amino acid residues)**
plyBt33-F/ *Bam*HI	GAGGATCC^*^ATGGGTTACACTGTAGATATTTC	*plyBt33* (816bp)	PlyBt33/33kDa (amino acid residues 1–272)
plyBt33-R/ *Sal*I	GACGTCGACTTCTTTTGTATAAAAGTATTTAA		
plyBt33-F/ *Bam*HI	GAGGATCCATGGGTTACACTGTAGATATTTC	*plyBt33-N* (558bp)	PlyBt33-N/24kDa (amino acid residues 1–186)
plyBt33-N-R/ *Sal*I	GACGTCGACTGTAAACCAATCTAACGACT		
plyBt33-IC-F/*Bam*HI	GAGGATCCCTTGGATACACTTCAAAAAT	*plyBt33-IC* (258bp)	PlyBt33-IC/11kDa (amino acid residues 187–272)
plyBt33-R/ *Sal*I	GACGTCGACTTCTTTTGTATAAAAGTATTTAA		

^*^The characters underline represents the restriction enzymes digest sites.

### Protein expression and purification

Three transformants containing genes *plyBt33*, *plyBt33-N*, and *plyBt33-IC* were cultured in LB broth containing 100 μg/ml ampicillin at 37°C with moderate rotation until cultures reached OD_600_ = 0.4. Cultures were then induced by the addition of 1 mM IPTG at 16°C for 4 h. Cells were collected by centrifugation at 10,000 × *g* for 10 min and resuspended in 20 mM Tris-Cl (pH 7.5). Following ultrasonication, debris was removed by centrifugation and the suspensions were harvested. Following filtration, proteins in the suspensions were purified using a Ni-nitrilotriacetic acid (NTA; Qiagen, German) column according to the manufacturer’s instructions. Proteins PlyBt33 and PlyBt33-N were analyzed using 10% SDS-PAGE, while protein PlyBt33-IC was analyzed using 15% SDS-PAGE. Protein concentrations were calculated using the Bradford method [45]. Purified proteins were dialyzed against 20 mM Tris-HCl (pH 8.0) and stored at −20°C until required.

### Lytic activity assay

Crude protein extracts and purified proteins were assayed for lytic activity as described previously [7,17]. *B. thuringiensis* strains HD-73 and HD-1, four *B. thuringiensis* isolates, *B. subtilis*, *B. pumilus*, *B cereus, B. anthracis,* and the Gram-negative strains *P. aeruginosa, Y. pseudotuberculosis*, and *E. coli* were used as indicator strains*.* Strains were grown to mid-exponential phase in LB broth, and then cells were harvested by centrifugation and resuspended in 20 mM Tris-HCl buffer (pH 8.0). The Gram-negative strain cells were treated with 1 mM EDTA in PBS to permeabilize the outer membranes prior to testing their susceptibility to PlyBt33. For rapid screening of the lytic spectrum, the indicator strains were plated onto LB plates and crude lysate of expressed proteins was added to filter paper that was placed on the bacterial lawn. Plates were incubated at 30°C overnight. Additionally, purified proteins were added at a ratio of 1:9 to cell suspensions (initial OD_600_ = 0.8) and the absorbance at OD_600_was monitored at 37°C for 1 h with a multimode reader (Bio-Tek Synergy HT, Winooski, VT). The crude extract of *E. coli* M15 containing plasmid pQE-30 was used as a control.

The optimal reaction pH of the endolysin was examined by adding dialyzed endolysins to cells of *B. thuringiensis* strain HD-73 resuspended in a series of buffers (20 mM Tris) at various pH levels (pH 3.0, 4.0, 5.0, 6.0, 7.0, 8.0, 9.0, 10.0, 11.0, and 12.0), and the OD_600_ was monitored as described above. The optimal reaction temperature of the endolysin was tested in 20 mM Tris-HCl (pH 8.0) at temperatures of 10–80°C in 10°C increments, and the OD_600_ was again monitored. To analyze the endolysin thermostability, endolysins in 20 mM Tris-HCl (pH 8.0) were first treated at different temperatures (4°C, 30°C, 40°C, 50°C, and 60°C) for 1 h and the lytic activity was tested as described above. All experiments were carried out in triplicate.

### Labeling and binding activity assay of PlyBt33-IC

To test binding activity, purified PlyBt33-IC was labeled with FITC (Sigma-Aldrich, Saint Louis, MO) according to the manufacturer’s instructions. Following purification, PlyBt33-IC protein was dialyzed four times against FITC reaction buffer (7.56 g NaHCO_3_, 1.06 g Na_2_CO_3_, 7.36 g NaCl, with MilliQ water added to 1 l and the pH adjusted to 9.0). FITC was dissolved in dimethyl sulfoxide to a concentration of 1 mg/ml and added into the PlyBt33-IC suspension at a ratio of 150 μg FITC to 1 mg PlyBt33-IC. Following 8 h incubation at 4°C in the dark, the reaction was stopped with NH_4_Cl at a final concentration of 50 mM for 2 h at 4°C in the dark. The labeled protein was dialyzed against PBS (8 g NaCl, 0.2 g KCl, 3.49 g Na_2_HPO_4_.12H_2_O, 0.24 g KH_2_PO_4_, with MilliQ water added to 1 l and the pH adjusted to 7.4) several times until the dialysis liquid was colorless, and stored at −20°C until required. BSA was also labeled as above and used as a control. FITC-labeled proteins were named FITC-PlyBt33-IC and FITC-BSA.

The specific binding activity of PlyBt33-IC to the cell wall was assayed as described previously with some modifications [12,46]. *B. thuringiensis* strain HD-73 was grown to mid-exponential phase in LB broth (OD_600_ = 0.8), and the cells were harvested by centrifugation (10,000 × *g* for 1 min) and resuspended in a one-tenth volume of PBS-T (pH 7.4, 0.01% Tween 20). FITC-labeled PlyBt33-IC was added to a 100 μl cell suspension to a final concentration of 0.0125 mg/ml and incubated at 30°C for 5 min. For fluorescence microscopy observation, the cells were harvested by centrifugation and washed twice with 500 μl PBS-T buffer. The pellet was then resuspended in 50 μl PBS-T. FITC-labeled BSA was used as a control. All cells were observed using an Olympus BX51 microscope.

## Competing interests

The authors have no competing interests to declare.

## Authors’ contributions

YHY and QP conducted the protein analysis. YHY performed the bioinformatics analyses. MYG supervised the work. MYG and YHY designed the study and wrote the manuscript. All authors reviewed and approved the final version of the manuscript.
